# The impact of adoption of a new urate-lowering agent on trends in utilization and cost in practice

**DOI:** 10.1371/journal.pone.0221504

**Published:** 2019-08-26

**Authors:** Yueh-Lung Peng, Chien-Te Lee, You-Lin Tain, Yaw-Bin Huang, Hung-Yi Chuang, Yen-Hsia Wen, Shiou-Huei Huang, Chun-Yu Tsai, Chien-Ning Hsu

**Affiliations:** 1 School of Pharmacy, Kaohsiung Medical University, Kaohsiung, Taiwan; 2 Division of Health Technology Assessment, Center for Drug Evaluation, Taipei, Taiwan; 3 Department of Internal Medicine, Division of Nephrology, Kaohsiung Chang Gung Memorial Hospital, College of Medicine, Chang Gung University, Kaohsiung, Taiwan; 4 Department of Pediatrics, Division of Pediatric Nephrology, Kaohsiung Chang Gung Memorial Hospital, College of Medicine, Chang Gung University, Kaohsiung, Taiwan; 5 Department of Public Health and Center of Excellence for Environmental Medicine, Kaohsiung, Taiwan; 6 Department of Pharmacy, Kaohsiung Chang Gung Memorial Hospital, Kaohsiung, Taiwan; 7 Department of Pharmaceutical Materials Management, Chang Gung Memorial Foundation, Taoyuan, Taiwan; Anahuac University Mexico, MEXICO

## Abstract

**Objectives:**

Changes in treatment choice of therapy and size of treated population that can lead to under- or overestimate of payer’s budget are less likely to be reassured after reimbursement adoption of a new drug. The aim of this study was to evaluate the effects of febuxostat introduction and the modifications in its insurance coverage on the utilization and expenditure of urate-lowering therapy (ULT).

**Methods:**

Electronic medical records for adults patients prescribed any ULT during 2010–2015 was derived from the largest medical organization in Taiwan. Aggregated estimates of ULT use and costs were assessed per 3-month and per patient per month (PPPM). Factors associated with total ULT expenditure were assessed using a time series design with factored Autoregressive Integrated Moving Average (ARIMA) models.

**Results:**

ULT prevalent users increased 34.1% from 2010 to 2015 and a 123% increase in total ULT expenditure. Numbers on allopurinol and sulfinpyrazone both declined 31%, and on benzbromarone and febuxostat gradually increased to 38.21% and 22.89% of all users in 2015. Insurance payments PPPM ($4.44 to $9.22) and total monthly ULT cost ($32,946 to $ 85,732) growth more than doubled in 6 years, trend changes generated mostly by individuals switching to febuxostat.

**Conclusions:**

ULT use moved to favor benzbromarone and febuxostat; greater expensive uptake for febuxostat led to a rapid rise in ULT cost. Marginal values of increasing access to febuxostat for asymptomatic hyperuricemia should be focus on future studies to facilitate drug prices negotiation and ensure appropriate ULT use.

## Introduction

The global prevalence of gout and hyperuricemia have been increasing, in part due to the growing availability of high caloric foods and the rising prevalence of comorbidities that increase risk of hyperuricemia, such as hypertension, obesity, metabolic syndrome, and chronic kidney disease (CKD) [1]. Gout is associated with a substantial economic burden: the annual direct costs of new cases of acute gout in the United States (US) may be as high as US$ 27 million [2]. Individuals who experience attacks of gout also exhibit greater absenteeism, which may lead to reduced productivity and an impaired health-related quality of life [3,4]. These observations highlight the need for an efficient strategy for the management of hyperuricemia in the healthcare system.

Treatment with urate-lowering therapy (ULT) to lower serum uric acid (SUA) is recommended for patients with symptomatic gout, joint damage, and/or severe hyperuricemia [5–7], but there is a lack of direct evidence to support the treatment of asymptomatic patients [8]. There have been recent advances in the treatment of hyperuricemia: febuxostat, a new xanthine-oxidase inhibitors (XOI), became available worldwide in 2009 and was found to be significantly more effective in lowering uric acid levels than allopurinol in trial settings [9,10]. Although allopurinol (XOI) is generally well tolerated and effective in most individuals, its utilization has gradually declined due to associations with severe cutaneous adverse reactions (SCARs) in those with specific genetic markers (human leukocyte antigen [HLA]-B*58:01) and the risk of SCARs increased among individuals with impaired kidney function [11].

In 2011, the national health insurance (NHI) program in Taiwan covered febuxostat as second line therapy for patients who failed to respond or could not tolerate the first-line regimen of allopurinol and uricosuric agents. On 01 March, 2014, the NHI policy on prescription drugs was modified to cover the use of febuxostat as first-line therapy for patients with moderate CKD (estimated glomerular filtration rate [eGFR] <45 ml/min/1.73 m^2^ or serum creatinine >1.5 mg/dL) [12]; this was further expanded to those diagnosed with uric acid nephrolithiasis on 01 August, 2016 [13]. The reimbursed daily price of febuxostat (80 mg/table/day, US$ 0.75) under the Taiwan NHI program in 2016 was two to eight times higher than allopurinol (100 mg/table, 300mg/day, US$ 0.14), and uricosuric agents: benzbromarone (50 mg/tab, 100mg/day, US$ 0.09) and sulfinpyrazone (100mg/tab, 300 mg/day, US$ 0.37) [14].

The effects on healthcare spending of changes in insurance coverage of innovative prescription drugs have previously been described in different disease populations [15,16], yet the aggregate demand for prescriptions associated with coverage expansions and therapeutic substitutions in management of hyperuricemia and gout are not well understood. We conducted a cross-sectional study to evaluate how the adoption of febuxostat and the modifications in its insurance coverage have affected trend changes in the ULT use and expenditure in a large Taiwan medical organization.

## Methods

### Study design and patient population

This was a cross-sectional study using electronic medical records from Chang-Gung Memorial Hospital (CGMH), the largest medical center in Taiwan. CGMH provides approximately 10–12% of healthcare services of the Taiwan NHI program. The CGMH database contains diagnostic, prescription, and laboratory test results from both in- and outpatient settings. Individuals ≥18 years of age who had been prescribed at least one ULT, including febuxostat, allopurinol, benzbromarone, probenecid, or sulfinpyrazone, between 01 January 2010 and 31 December 2015 were identified. This study was approved by the Institutional Review Board of CGMH and the Ethics Committee of CGMH, Taiyuan, Taiwan (approval number 201600110B0). All data were anonymized to protect participant confidentiality.

### Patterns of utilization

The primary unit of analysis was person-ULT prescription, referred to as individual ULT users during the study period. For a single patient encounter, drug mentions are duplicated by the number of different ULT is prescribed. The proportion of ULT users was calculated from the number using each urate-lowering agent divided by the total number of individuals using ULT in each 3-month interval (quarter). An individual was considered to be using a ULT if they had been prescribed the drug for ≥7 days in each quarter. Those who received therapy for <7 days were excluded because they were thought likely to have been prescribed the drug at an acute stage, or were less likely to use it long-term. To assess the effects of expansion of febuxostat insurance coverage on patterns of ULT use, febuxostat users were categorized as either a *switcher* or a *new user*. Febuxostat switchers were those who have been treated with allopurinol or any uricosuric agents. A new user was an individual who had newly initiated febuxostat and had not previously been prescribed a ULT. Patient’s age at the ULT prescribed and sex were analyzed. The physician’s specialty for each ULT prescriptions was assessed to explore the correlation between febuxostat adoption and specialty in the study setting.

### Calculation of expenditures

Trends in ULT expenditure were measured for all ULT users and for individual patients with individual ULT use per month. ULT expenditure per month and per 3-month were calculated by dividing the sum of ULT costs by the total number of months participants were prescribed with a ULT. For individual patients, ULT cost per patient per month (PPPM) was calculated by summing paid for any ULT divided by month of exposure. All cost estimates were normalized to September 2016 US dollars using the amount paid by the Taiwan NIH program. The exchange rate for 1 US dollar was 31.5 TWD in September, 2016.

The amount paid for each ULT per tablet was retrieved from the reimbursed products file on the National Health Insurance Administration webpage, [14] which reports the time of coverage and amounts paid by the prescription billing code. To account for the variation in reimbursed price over time, the national annual average cost per unit was calculated based on the products with the same strength and same dosage form that were reimbursed in the same period of time (S1 Table).

### Statistical analysis

Cochran–Armitage tests were performed to examine for statistical significance in trends of each and all ULT uses over the study period. Observed incidence and prevalence and demographic changes can be used in a simulation model to predict future overall healthcare costs and the financial impacts in the target population [15]. Time series modeling techniques for forecasting the future value, describing the pattern of change in a variable, and assessing the impacts of event have been increasing used in drug utilization research [16,17]. The impacts of febuxostat introduction to the study setting from 01 April, 2013 (intervention 1) and expansions of febuxostat insurance coverage from 01 March, 2014 (intervention 2) on longitudinal trends in total all ULT expenditure (including febuxostat and its alternatives) were assessed using the autoregressive integrated moving average (ARIMA) model to adjust for seasonality and autocorrelation in the serial correlation. Due to the linear time-independent hypothesis model is usually not suitable to examine the trend changes in drug utilization throughout a period of time, prior studies have suggested that segmented regression analysis is preferable to assess the longitudinal impact of an event [16]. Attacks of gout have been noticed to be seasonal [18], so ARIMA regression model was employed to account for possible seasonality bias and assess intervention effects on the trend changes in ULT expenditure.

Briefly the independent variables in the statistical model were fitted first, followed by the ARIMA modeling identification process to the residuals [19]. The factored ARIMA (p, q) model with intercept was used in this study, where parameters p (autoregressive part) and q (moving average part) were non-negative integers (range from 0 to 12) in the model and a maximum likelihood method was used to assess the effect of the interventions on total ULT expenditure. The autocorrelation, partial autocorrelation, and inverse autocorrelation correlation plots were assessed for model parameter selection and appropriateness. We used the estimated rates generated by the Factored ARIMA Model Specification window for the change in total ULT expenditure [20]. In the time series intervention analysis, the slope coefficient measures the relative change in the dependent variable (total ULT expenditure) for a given absolute change in the value of the explanatory variable (as intervention 1, 2) at month t. The coefficients of intervention 1 and 2 indicate the monthly changes in ULT expenditure associated with febuxostat introduction or reimbursement coverage activation calculated in the ARIMA model. All analyses were conducted using SAS 9.3 (SAS Institute, Cary, North Carolina, USA); *p*-values of <0.05 were considered significant.

## Results

### Patients

**Fig 1:**
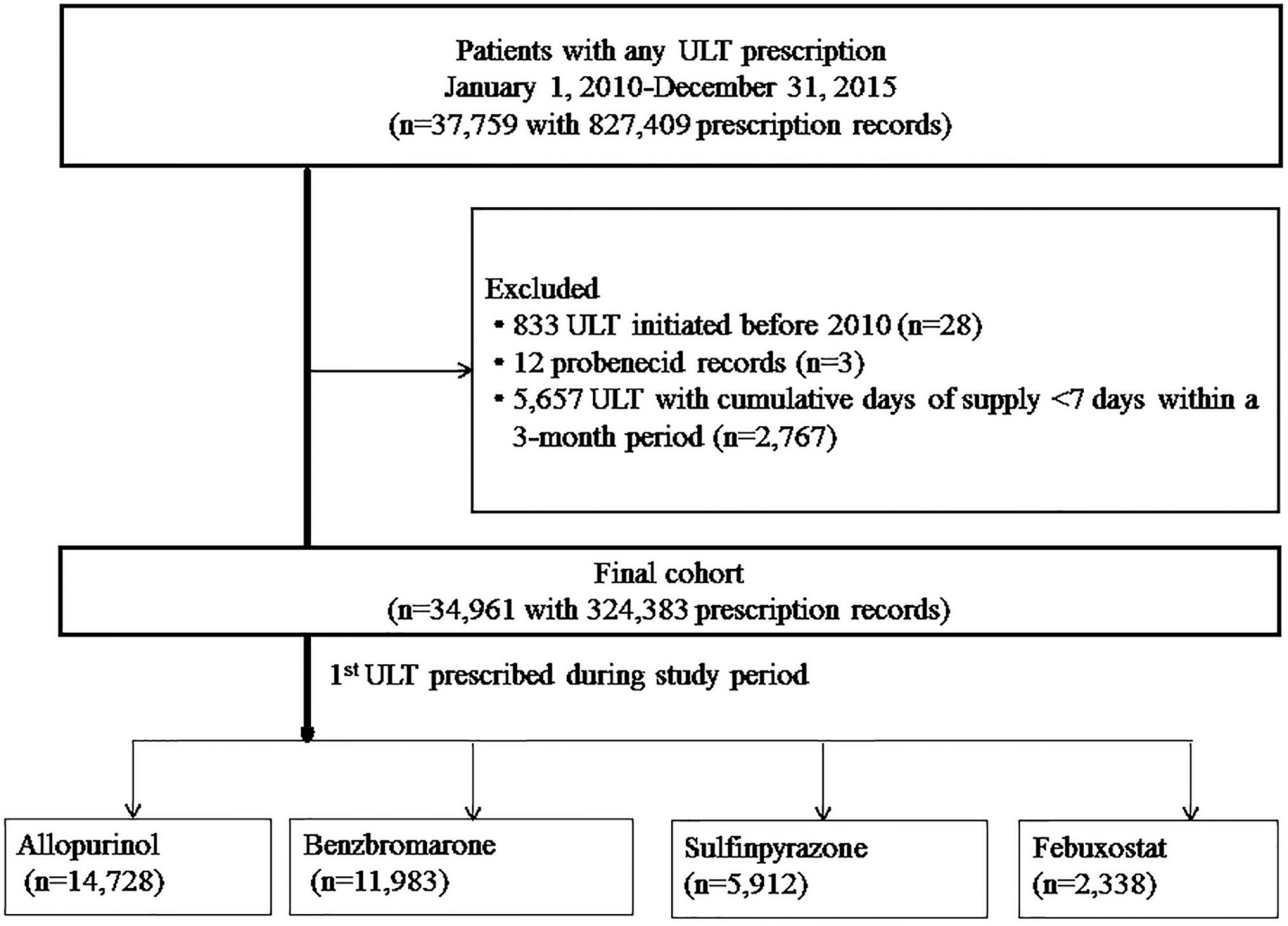
Selection of the study cohort.

Between 2010 and 2015, 37,759 patients were prescribed at least one ULT (Fig 1); 34,963 (92.6%) were prescribed the drug(s) for ≥7 days. The primary unit of analysis was person-ULT prescription. The n denotes the number of patients prescribed the ULT in the study period. Three individuals were prescribed probenecid (<10 per 100,000 persons) and were therefore excluded from further analyses. Of the 34,961 individuals prescribed ULT (for 324,383 prescription orders), 14,728 (42.1%) were initially prescribed allopurinol, 11,983 (34.3%) benzbromarone, 5,912 (16.9%) sulfinpyrazone, and 2,338 (6.7%) febuxostat during the study period (Table 1). Overall mean age at first recorded prescription was 62.2 (standard deviation [SD] ± 15.8) years; this was slightly higher in febuxostat users (64.3±14.8 years). A total 27,083 (77.5%) participants were male. Overall, participants were most frequently prescribed ULT by cardiologists (7,665/34,961 [21.9%]), followed by nephrologists (6,490/34,961 [18.6%]), rheumatologists (6,375/34,961 [18.2%]), and neurologists (3,232/34,961 [9.2%]). The distribution of specialists varied depending on the drug prescribed.

**Table 1:** Characteristics of users by the first prescription of urate-lowering agent during the study period.

	All users (n = 34,961)	Allopurinol (n = 14,728)	Benzbromarone (n = 11,983)	Sulfinpyrazone (n = 5,912)	Febuxostat (n = 2,338)
Age, mean (SD), years	62.18 (±15.77)	61.53 (±16.24)	62.14 (±15.56)	63.04 (±15.25)	64.32 (±14.83)
Sex, %					
Male	27,083 (77.47%)	11,595 (78.73%)	9,252 (77.21%)	4,588 (77.60%)	1,648 (70.49%)
Female	7,878 (22.53%)	3,133 (21.27%)	2,731 (22.79%)	1,324 (22.40%)	690 (29.57%)
Specialty, %					
Cardiology	7,665 (21.92%)	2,472 (16.78%)	2,981 (24.88%)	1,981 (33.51%)	231 (9.88%)
Nephrology	6,490 (18.56%)	3,116 (21.16%)	1,389 (11.59%)	670 (11.33%)	1,315 (56.24%)
Rheumatology	6,375 (18.23%)	2,549 (17.31%)	2,557 (21.34%)	1,017 (17.20%)	252 (10.78%)
Neurology	3,232 (9.24%)	836 (5.68%)	1,500 (12.52%)	862 (14.58%)	34 (1.45%)
Endocrine	3,119 (8.92%)	1,168 (7.93%)	1,202 (10.03%)	636 (10.76%)	113 (4.83%)
Hematology/oncology	1,051 (3.01%)	838 (5.69%)	166 (1.39%	3 (0.05%)	44 (1.88%)
Internal medicine	941 (2.69%)	361 (2.45%)	237 (1.98%)	304 (5.14%)	39 (1.67%)
Urology/Surgery	934 (2.67%)	424 (2.88%)	462 (3.86%)	5 (0.08%)	43 (1.84%)
Family medicine	757 (2.17%)	281 (1.91%)	246 (2.05%)	214 (3.62%)	16 (0.68%)
Others	4,397 (12.58%)	2,683 (18.22%)	1,243 (10.37%)	220 (3.72%)	251 (10.74%)

All ULT users in use trends analyses were patients had been prescribed the individual ULT for ≥7 days in each quarter between 2010 and 2015

### Adoption of febuxostat

**Fig 2:**
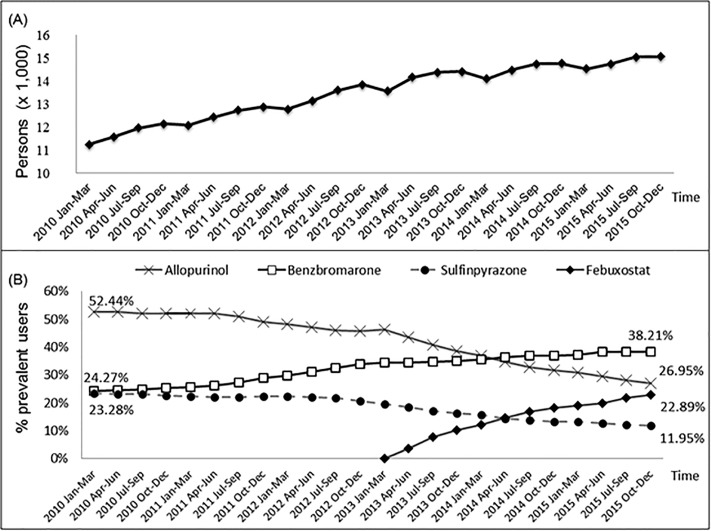
A.Cumulative numbers of patients using ULT with distribution over 3-month intervals from 2010–2015. Cochrane-Armitage trend test, P<.0001; B.Cumulative numbers of patients using individual ULT displayed as proportions of the total number of ULT users, with distribution over 3-month intervals from 2010–2015.

The number of individuals using ULT increased from 11,235 to 15,069 (34.1% increase) during the study period (Cochrane-Armitage trend test, *p*<0.0001; Fig 2(A)) and there were 15,069 patients in the last quarter of 2015 (2015 Q4). At the beginning of 2010, 52.4% of those using ULT were on allopurinol (n = 5, 892), 24.3% on benzbromarone (n = 2,727), and 23.3% on sulfinpyrazone (n = 2,616) (Fig 2(B)). By the end of 2015, this had reduced to 27.0% for allopurinol (n = 4,061), increased to 38.2% for benzbromarone (n = 5,758).

After the introduction of febuxostat in April 2013, its usage increased rapidly to 22.9% (n = 3,450) by the end of 2015. The increase in use of febuxostat was higher among switchers than new users (15.1% vs. 7.8%). The proportion of individuals prescribed febuxostat remained higher in switchers than new users after the expansion of febuxostat coverage to patients with CKD enacted on 01 March, 2014.

Of the 6,133 individuals using febuxostat, 3,795 (61.9%) were switchers and 2,338 (38.1%) were new users (Table 2). The mean age was higher in new users than in switchers (64.3±14.8 vs. 61.2±15.0 years). More than half of new users (n = 1,315) received treatment from nephrologists; switchers were primarily treated by nephrologists (39.8%), rheumatologists (20.3%) and cardiologists (13.1%).

**Table 2:** Annual total costs of all ULT and individual ULT proportional cost in each year.

Year	Total costs (USD)	Incremental rate	Benzbromarone	Allopurinol	Sulfinpyrazone	Febuxostat
2010	403380.17		16.91%	27.27%	55.82%	-
2011	411143.33	1.93%	18.03%	27.06%	54.68%	-
2012	419337.03	3.96%	18.32%	29.25%	52.94%	-
2013	538361.64	33.46%	15.60%	21.69%	35.34%	27.31%
2014	774567.50	92.02%	12.13%	14.18%	18.38%	55.40%
2015	901342.81	123.45%	9.38%	11.92%	12.83%	65.00%

The incremental rate in each year was determined by the change in annual total costs from 2010 divided the annual total costs in 2010. For each year, individual ULT proportional cost was based on the summed cost of individual ULT divided by the total costs of all ULT in the given year.

The exchange rate for 1 US dollar was 31.5 Taiwan dollars (TWD) in September, 2016 for patients ever prescribed with any ULT during the study period.

### ULT expenditure

The annual expenditure for ULT increased from US$ 403,380 in 2010 to US$901,343 in 2015 (123% increase; Table 2). The proportion of ULT expenditure spent on febuxostat grew from 27.3$ (US$ 147,014) in 2013 to 65% (US$ 585,911) in 2015 of all users. Proportions spent on sulfinpyrazone and allopurinol between 2010 and 2015, however, decreased from 55.82% (US$225,155) to 12.83% (US$115,665) and from 27.3% (US$110,017) to 12% (US$107,470); respectively. Spending on benzbromarone showed an increase (24%) between 2010 and 2015 (from US$ 68,208 to US$ 84,519), and its proportion of annual total cost declined from 16.91% to 9.38%; respectively.

**Fig 3:**
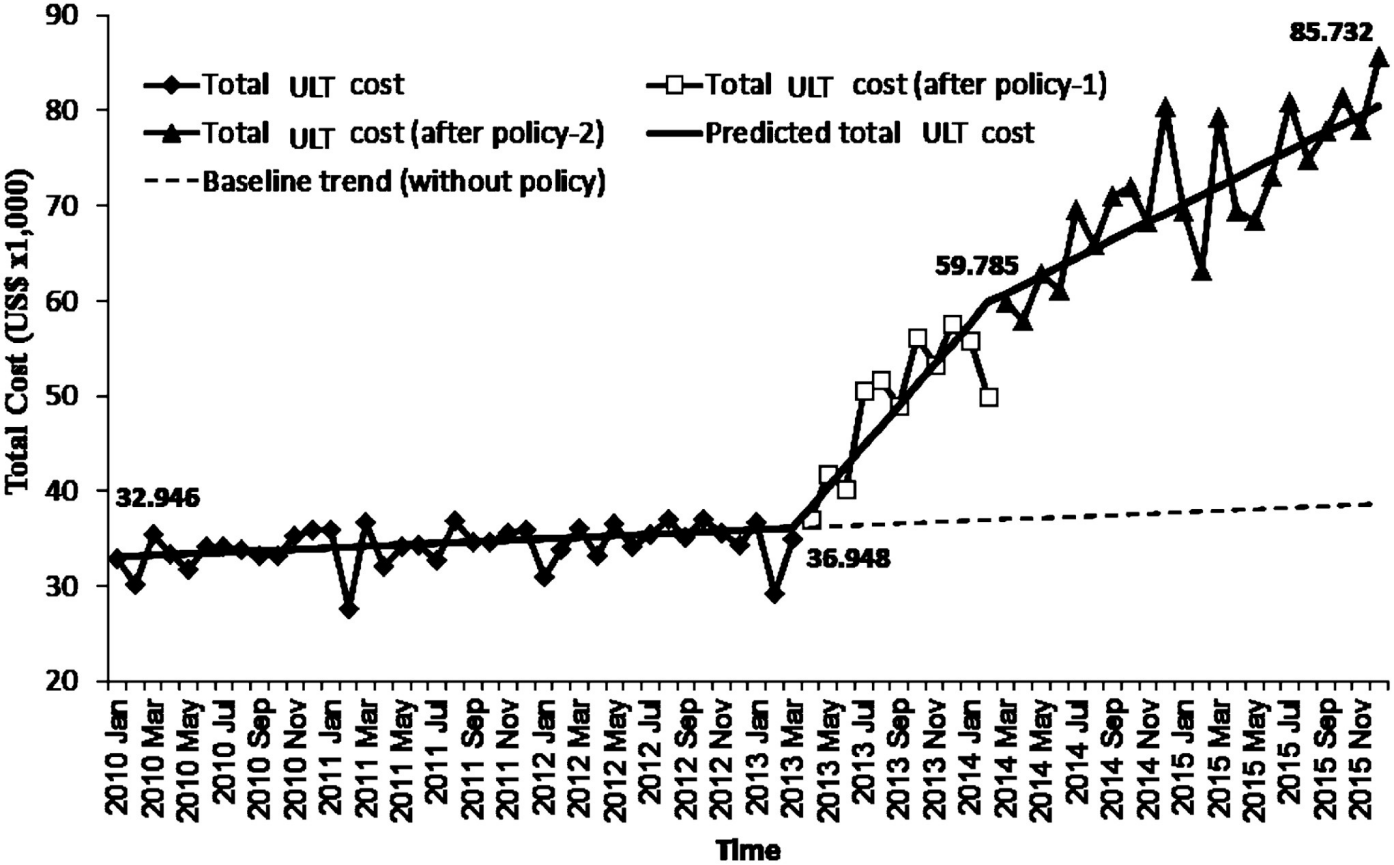
Total spent per month on ULT in the study setting from January 2010 to December 2015 (US dollars). Policy 1: introduction of febuxostat, policy 2: expanded insurance policy for febuxostat as the first-line therapy for CKD stage 3 and higher stages.

The aggregated total spent on ULT per month increased from US$ 32,946 in January 2010 to US$ 85,732 in December 2015 (160% increase; Fig 3). The factored ARIMA model indicated that the inclusion of febuxostat was associated with an increase in the total cost of ULT (*p*<0.0001; Table 3). Although including febuxostat as first line therapy for CKD was associated with a significant small decrease in monthly total cost, this did not offset a slow and steady increase (43% increase) after March, 2014 (*p*<0.0001; Table 3) in Fig 3.

**Table 3:** ARIMA regression-predicted change in total monthly ULT expenditure.

Dependent variables	Factored ARIMA model	Independent variables	Coefficients	*p* value
Drug Expenditure (unit: US$)	p = (12); q = (3)	Intercept	32982.4	<.0001
		Baseline trend	79.15	0.12
		Trend change 1	2081.3	<.0001
		Trend change 2	-1220.6	<.0001

ARIMA model = autoregressive integrated moving average model; model parameter: p = autoregressive order, d = differencing (the default value, 0) and q = moving average order, Trend change 1: indicate the febuxostat adoption, Trend change 2: indicate the expansion of febuxostat coverage policy for patients with chronic kidney disease

PPPM for ULT increased from US$ 4.44 (±4.79) to US$ 9.2 (±12.43) from January 2010 to December 2015 (107% increase). PPPM for febuxostat was higher in switchers (US$ 23) than new users (US$19.5) in April 2013 and increased 16% (US$ 26.8) and 9% (US$ 21.3) to December 2015, respectively.

The exclusion criterion was a patient who dispensed an ULT less than 7 days in a 3-month interval. For 2,767 patients (5,657 prescription records: allopurinol (n = 2,752), 48.65%; benzbromarone (n = 1,804), 31.89%; sulfinpyrazone (n = 533), 9.42%; and febuxostat (n = 568), 10.04%) not included in the analysis. Over 92% of excluded patients prescribed with an ULT one time only in the entire study period. Total cost of exclusion for intermittent uses was US$ 994.87 (<0.04% annual cost of ULT over time) deemed no significant effect on the trend changes in total ULT expenditure.

## Discussion

ULT are increasingly used in the clinical setting and impose a growing financial impact on the health care system, particularly resulting from the addition of febuxostat. Our results demonstrate the annual ULT payment increased by 123% between 2010 and 2015, with an increase of 34.1% in the number ULT users. The major driver of a large increase in use and cost was 61.9% febuxostat switchers. Although the increased use of febuxostat may represent therapeutic substitution for greater effectiveness, it may also be explained by a need to receive the newest drug available, or as the consequences of changes to insurance policy regarding prescriptions covered.

A numerous studies have consistently demonstrated a link not only between hyperuricemia and gout flares, but also between hyperuricemia and CKD progression, cardiovascular disease [21–23], and higher all-cause mortality rates. [24] However, there is lack of direct evidence to support or refute the use of ULT to lower SUA in patients with CKD and symptomatic or asymptomatic hyperuricemia for CKD prevention. The Kidney Disease: Improving Global Outcomes (KDIGO) clinical practice guideline recommends that CKD patients be encouraged to adopt lifestyle and dietary management strategies rather than use ULT. [25] The use of HLA-B*58:01 genotyping prior to allopurinol administration could be justified, as part of a cost-effectiveness strategy to avoid allopurinol-related SCARs [26]. However, instead of waiting for genotyping results, physicians may choose febuxostat as first-line therapy to avoid allopurinol-related hypersensitivity syndrome in particular for patients with CKD.

However, we found that the choice of ULT prescribed by a physician may simply depend on an individual’s comorbidities, the severity of hyperuricemia, and on the accessibility of the newest drug (e.g., febuxostat). Patient characteristics in our study cohort were comparable with gout patients with and without CKD in a cohort in the US initiating allopurinol and febuxostat [27,28]. Patients receiving ULT were cared for mainly by cardiologists, nephrologists, and rheumatologists, reflecting the pattern of comorbidities seen in our clinical setting. Individuals who switched to febuxostat after using allopurinol, benzbromarone, or sulfinpyrazone were common (61.9%), and more than half of febuxostat new users were treated by nephrologists. This is consistent with a study by Kim et al [27], who found that individuals prescribed febuxostat were more likely to have a previous diagnosis of CKD (odds ratio [OR] 2.18, 95% confidence interval [CI] 1.88–2.52) or hypertension (OR 1.11, 95% CI 1.01–1.22) [27]. Over half (52%) of these individuals had previously been treated with allopurinol.

This study also shows that benzbromarone accounted for 24% of the increase in use among all users, but only for about 9.38–18.32% of total spending on ULT over the study period. The policy intervention for the wider use of febuxostat as first-line therapy in patients with CKD stage 3 showed a smaller effect on the rising trend of total ULT spending (trend change -1220.6, *p*<0.0001) than the introduction of use. The slight reduction in switchers was observed in 2015 (S1 Fig), when the total cost of ULT steadily increased. Possible explanations may be that the reimbursed price of febuxostat declined by a 3.7% 2014 price per unit tablet, or hospital-based budgets led to the observed decrease in individuals switching to and being initiated on febuxostat. Furthermore, physicians may have been aware of a new major febuxostat-related hypersensitivity report [29], and of cardiovascular safety concerns that have arisen in 2014/2015, to the extent that this knowledge discouraged the use of febuxostat.

Budget impact analysis (BIA) has been proposed to assess the financial consequences of adopting a new intervention [30]. A cohort of 980,000 individuals with gout was employed in a BIA in 2011 with about 40–44% patients receiving any ULT; the market share was estimated to be 5.5% the in the first year post-febuxostat market and increased to 22% in the fifth year [31]. This resulted in an aggregated budget for febuxostat of US$ 1.11 million (TWD 33 million) and US$5.71 million (TWD 169 million) in 2011 (1 USD = 29.6 TWD) for the first and fifth year, respectively. Estimates were generated of the budgetary impact of febuxostat used as a second-line agent for patients with symptomatic hyperuricemia who have exhibited intolerance or failed to respond from prior therapy of allopurinol, benzbromarone, sulfinpyrazone, or probenecid, based on primary Taiwan FDA-approved indications.

Our data show that febuxostat dominated 27.3%, 55.4% and 65% of total ULT costs in the first, second, and third year, respectively, after its introduction, with an increase in total costs of 123% over a 6-year period and of 67% increase after the introduction of febuxostat in 2013. These results suggest that the budgetary impact of febuxostat was severely underestimated when compared with real-world treatment paradigms and the diffusion rate of febuxostat in the first 3 years of availability.

Our observations are confirmed by a recent BIA that estimated the impacts on pharmacy and medical costs of the broader adoption of febuxostat as first-line gout therapy for individuals with and without CKD, based on a US payer perspective [32]. The overall febuxostat market share was assumed at 18%; under 30% of individuals with gout were assumed to receive an XOI in the model [32]. The study showed that the cumulative budget impact of febuxostat usage on treatment costs (drugs and SUA tests) increased from 65.8% to 128.3% over a 3-year period. These results suggested that clinical practice paradigm should be taken into accounted in estimating the financial impacts associated with ULT in the healthcare system [30].

The recent trend in utilization of ULT has not been robustly examined following the introduction of febuxostat in the clinical setting until now. Current empirical evidence suggest that the adoption of high price of febuxostat and its insurance coverage expansion were major driving force led to changes in treatment patterns which increased total ULT cost. This highlights the critical need for the use of value-based reimbursement policies to curb the escalating costs of ULT. Price negotiation, such as price-volume agreements [33], and value-based pricing [34] may also have an impact on lower drug spending. However, an appropriate price negotiation scheme requires available evidence to negotiate. Further comparative effectiveness research is warranted for long-term outcomes in individuals with asymptomatic hyperuricemia, particularly for those with multiple comorbidities who are at higher risk of hyperuricemia-related CKD progression, cardiovascular events, and all-cause mortality.

The results of the present study need to be interpreted in the context of study limitations. First, the unit of analysis is a hospital level aggregation of prescriptions. The major ULT users aged>65 years old (mean age, 62.18 (±15.77)) is roughly consistent with a high prevalence of gout after age 60 years in the Taiwan population [35]. In addition, the study results show that patients had comorbid conditions with cardiovascular and kidney disease were more obvious in the case of therapeutic substitution, which coincided with this period of overall findings in patterns of ULT use derived from the U.S. population [27,28,32]. Further, the ARIMA method has been proven to be very useful to detect effect of a policy intervention on drug expenditure other than the underlying secular trend [36]. However, changing patient characteristics and risk factor profiles occurring at the same time as the study intervention could have affected the results of this study. Residual confounding may present and leads to under- or over-estimated level of trend changes in total ULT expenditure.

## Conclusions

The febuxostat rapid adoption in routine practice challenged previous budget impact estimation in the health care system sector. The observed shift towards the use of benzbromarone and febuxostat suggests a growing tendency to treat hyperuricemia, rather than gout. These findings underscore the effects of price and prescription drug insurance coverage on the ULT spending. Comparative effectiveness research is warranted to support financial protection for high-value therapies in preventing and slowing progression of hyperuricemia-related complications.

## Supporting information

S1 Fig Cumulative numbers of individuals prescribed febuxostat, stratified by switcher or new user status with distribution over 3-month periods. CKD stage 3 = chronic kidney disease with estimated glomerular filtration rate 45–30 ml/min/1.73m2.
(TIF)

S1 Table Annual average cost per unit of ULT applied in the analysis. (DOCX)

## Abbreviations

ARIMA: Autoregressive Integrated Moving Average
BIA: budget impact analysis
CI: confidence interval
CKD: chronic kidney disease
eGFR: estimated glomerular filtration rate
HLA: human leukocyte antigen
NHI: national health insurance
PPPM: per patient per month
SCARs: severe cutaneous adverse reactions
SUA: serum uric acid
ULT: urate-lowering therapy
US: United States (US)
XOI: xanthine-oxidase inhibitors
