# Supplementary material for: The impact of adoption of a new urate-lowering agent on trends in utilization and cost in practice
Source: PLoS One. 2019 Aug 26;14(8):e0221504. doi: 10.1371/journal.pone.0221504 (PMC6709886; doi:10.1371/journal.pone.0221504)
Supplement: S1 Table — (DOCX) [file pone.0221504.s002.docx]

Supplementary table 1 Annual average cost per unit of ULA applied in the analysis

| Year | Allopurinol 100mg/tablet | Benzbromarone 50mg/tablet | Sulfinpyrazone  100mg/table | Febuxostat  80mg/tablet |
| --- | --- | --- | --- | --- |
| 2010 | 1.03 (US$ 0.03) | 1.81(US$ 0.06) | 5.00 (US$ 0.16) |  |
| 2011 | 1.04 (US$ 0.03) | 1.77 (US$ 0.06) | 4.99 (US$ 0.16) |  |
| 2012 | 1.19 (US$ 0.04) | 1.43 (US$ 0.05) | 4.83 (US$ 0.15) | 25.90 (US$ 0.82) |
| 2013 | 1.19 (US$ 0.04) | 1.43 (US$ 0.05) | 4.83 (US$ 0.15) | 25.90 (US$ 0.82) |
| 2014 | 1.34 (US$ 0.04) | 1.51 (US$ 0.05) | 4.83 (US$ 0.15) | 25.37 (US$ 0.81) |
| 2015 | 1.50 (US$ 0.05) | 1.44 (US$ 0.05) | 4.05 (US$ 0.13) | 24.50 (US$ 0.78) |

Source: Costs for individual units of ULA are based on the reimbursed amount paid in Taiwan dollar by the National Insurance Administration in Taiwan at http://www.nhi.gov.tw/query/query1.aspx (in Traditional Chinese).

Because most ULA therapies are off-patent prescriptions, changes in unit costs are considered small during the study period. All cost estimates were normalized to September 2016 US dollars All cost estimates were normalized to September 2016 US dollars (1US dollar =31.5 Taiwan dollar).
